# Bacterial Metabolomics: Sample Preparation Methods

**DOI:** 10.1155/2022/9186536

**Published:** 2022-04-12

**Authors:** Khairunnisa Mohd Kamal, Mohd Hafidz Mahamad Maifiah, Nusaibah Abdul Rahim, Yumi Zuhanis Has-Yun Hashim, Muhamad Shirwan Abdullah Sani, Kamalrul Azlan Azizan

**Affiliations:** ^1^International Institute for Halal Research and Training (INHART), Level 3, KICT Building, International Islamic University Malaysia (IIUM), Jalan Gombak, Selangor 53100, Malaysia; ^2^Faculty of Pharmacy, University of Malaya, Kuala Lumpur 50603, Malaysia; ^3^Metabolomics Research Laboratory, Institute of Systems Biology (INBIOSIS), Universiti Kebangsaan Malaysia, UKM, Bangi, Selangor 43600, Malaysia

## Abstract

Metabolomics is a comprehensive analysis of metabolites existing in biological systems. As one of the important “omics” tools, the approach has been widely employed in various fields in helping to better understand the complex cellular metabolic states and changes. Bacterial metabolomics has gained a significant interest as bacteria serve to provide a better subject or model at systems level. The approach in metabolomics is categorized into untargeted and targeted which serves different paradigms of interest. Nevertheless, the bottleneck in metabolomics has been the sample or metabolite preparation method. A custom-made method and design for a particular species or strain of bacteria might be necessary as most studies generally refer to other bacteria or even yeast and fungi that may lead to unreliable analysis. The paramount aspect of metabolomics design comprises sample harvesting, quenching, and metabolite extraction procedures. Depending on the type of samples and research objective, each step must be at optimal conditions which are significantly important in determining the final output. To date, there are no standardized nor single designated protocols that have been established for a specific bacteria strain for untargeted and targeted approaches. In this paper, the existing and current developments of sample preparation methods of bacterial metabolomics used in both approaches are reviewed. The review also highlights previous literature of optimized conditions used to propose the most ideal methods for metabolite preparation, particularly for bacterial cells. Advantages and limitations of methods are discussed for future improvement of bacterial metabolomics.

## 1. Introduction

Systems biology is defined as an approach to provide valuable information on the fundamental biological questions of living systems [1]. The information is gained through a very dynamic process of integration and interaction of structural and functional complex [2]. With the advancement of scientific knowledge and technology, high-throughput measurements and analysis of various “omics” disciplines including genomics, transcriptomics, proteomics, lipidomics, and metabolomics are achievable [3]. The metadata is processed aiming for the reduction and integration of information and forming interconnected functional network systems [4, 5].

In the postgenomic era, in particular, metabolomics has been increasingly employed in various areas of research including food technology, plant, microbiology, drug discovery and development, metabolic engineering, and many more [6, 7]. Metabolomics, at a cellular level, provides an overview and snapshot of detailed characterization of significant metabolic changes of the phenotype [8]. The approach has been employed in refining the quality and safety of food products [9]. Metabolomics has been used in determining relationships and regulations between components of plant systems [10]. The tool also has provided important data to elucidate the pathophysiology of diseases and identify novel biomarkers making precision medicine feasible [11]. Microbial metabolomics, particularly the study of metabolic changes in bacteria, has gained significant research interest [12]. Bacterial systems provide robust functional information on its various biological functions [13]. The readily accessible databases on bacterial gene regulations and metabolic pathways enable the study of important bacterial systems including strain identification and differentiation [14], mechanism of action of drug [15–17], and metabolic changes upon antimicrobial treatment [18].

The approaches in metabolomics include untargeted and targeted depending on the research questions and objectives of the study [7, 19]. Untargeted metabolomics aims to uncover and identify all the possible cellular metabolites (known as metabolome) and metabolic changes [20]. Meanwhile, with targeted approach, definite or “targeted” known compounds are measured qualitatively and quantitatively to provide the detailed characterization of the metabolic enzymes and their kinetics [21]. A good metabolomics experimental design is incredibly important to produce reliable data and good correlation fitting with real physiological states or changes. Nevertheless, sample preparation method has been a major bottleneck in metabolomics study [22, 23]. A designated method would be necessary as bacterial cells exhibit dissimilar structure on their cell wall and membrane. Although various protocols on metabolite preparation have been developed, the optimized and standard method for a particular species of bacteria is still lacking. In addition, from the literature, information on metabolite preparation methods has not always been clearly described for untargeted or targeted approaches.

The analysis of metabolites requires advanced analytical instrument depending upon the untargeted or targeted approach. The processes involved are separation by chromatography technique (i.e., liquid chromatography (LC) and gas chromatography (GC)) and detection by spectrometer (i.e., mass spectrometry (MS)). Common analysis methods are nuclear magnetic resonance (NMR) spectroscopy, high performance liquid chromatography tandem-mass spectrometry (HPLC-MS/MS), and gas chromatography mass spectrometry (GC-MS). NMR and MS have been widely used for identification and quantification of a broad spectrum of metabolites [24]. NMR allows for the *in situ* determination of metabolite level but is limited to certain culture conditions or classes of metabolites [25]. LC-MS has been advantageously used over GC-MS for various types of samples as it offers dynamic coverage, range, high specificity, and much simpler sample preparation methods, while GC-MS is only suitable for volatile compounds [26]. Furthermore, recent technology of MS comes with additional platforms such as triple quadruple tandem MS (QQQ-MS/MS) and quadruple time-of-flight MS (QTOF-MS) resulting in better accuracies and sensitivities [27]. QQQ-MS excels in terms of specificity and sensitivity and thus is more favourable in targeted study; however, it has limitations in identifying unknown compounds [27, 28]. In contrast, QTOF-MS enables identification of a broad range of compounds with better accuracy and resolution, making it preferable in untargeted studies [27, 28].

In this review, metabolite preparation designs and methods from the last two or more decades are discussed to provide the overview on the methodological approaches used for bacteria sample. This review also highlights the optimized sample preparation methods of different bacterial species and strains according to untargeted and targeted approach. The scope of the discussion focuses on sample preparation methods for bacterial metabolomics only. Metabolite sample and data analysis are not covered in this review which are accessible in many existing literature.

## 2. Principles of Metabolomics

Metabolites are products of biochemical reactions, with low molecular weights of less than 1,500 Da, which exist inside (predominantly intracellular) and outside (extracellular) the cells [29]. Primary metabolic pathways involve both synthesis (anabolism) and breakdown (catabolism) of metabolome to produce building blocks and free energy, involving reactions of many enzymes with high turnover rates. Meanwhile secondary pathways are associated with stress responses and the intermediates are only used in conditions with limited growth rates and smaller number of reactions [30]. Comprehensive analysis of cellular metabolites and metabolic pathways can be executed via quantitative and qualitative methods [6, 31]. The levels or concentrations of metabolites exhibit direct relation of *in vivo* cellular biochemical activity and phenotype characteristics [29]. Therefore, any perturbations in metabolite levels induced by certain conditions are significant in understanding the whole cellular system of an organism or cells [21, 32, 33]. For instance, ATP and NADH are metabolites of primary metabolism which are highly sensitive upon exposure to conditions such as temperature and light which may result in degradation or being metabolized by enzymes [34].

The untargeted approach, also known as global metabolomics or global metabolic profiling, aims to profile all the intra- and extracellular metabolites available [11, 29]. All metabolites including the unknown are detected and identified putatively based on the existing databases. The analysis is then performed to study the relationship of the metabolic changes observed under specific treatments or conditions which are used to generate new hypothesis. The approach has been shown to successfully discover novel metabolites of interest, for instance, new diagnostic biological markers and potential antimicrobial compounds [12, 35–37]. In turn, targeted metabolomics is a hypothesis-driven approach where a predetermined set of compounds of a defined metabolic pathway of interest are analyzed for absolute identification and quantification [13, 21, 32]. Targeted approach is commonly driven after metabolites are identified from the earlier untargeted work. In-depth knowledge of particular metabolites and their specific pathway reactions would provide information on crucial metabolic enzymes and the kinetics and novel relationships between substrates and end products [38]. The results help to model metabolic pathway networks in predicting the outcomes of future biological experiments [15]. Targeted approach has been employed to uncover potential biomarkers [39], optimize drugs in targeting resistant bacteria [40], differentiate isogenic strains of bacteria [19], identify alterations of bacterial metabolic profiles upon antibiotic treatment [18], and predict and characterize antibiotics mode of action [16].

## 3. Experimental Design for Bacterial Metabolomics

**Figure 1:**
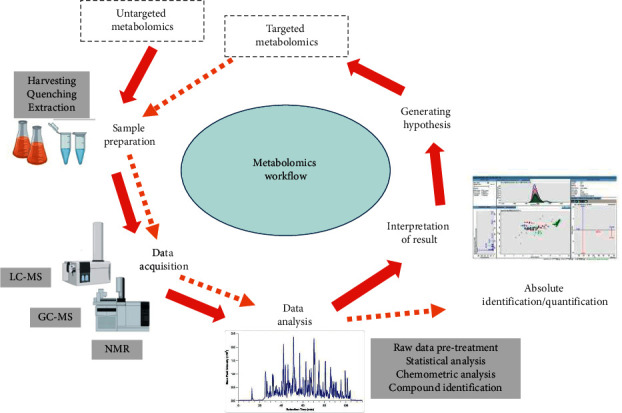
Overview of general metabolomics workflow. Solid (red) arrow represents the flow of untargeted metabolomics, while dotted (orange) arrow represents the flow of targeted metabolomics.

A clear research objective and question is important to direct the design of experiment to be either untargeted or targeted [29]. The process flow in metabolomics basically involves steps of sample pretreatment, sample analysis, pretreatment of raw data, statistical analysis, and finally the interpretation of results (Figure 1). Metabolite sample preparation method is the upmost important process and it is extremely sensitive as even minute changes will produce observable effects on the recovery of the types and levels of metabolites [30, 41–44]. The challenges may be due to the presence of many metabolites with high turnover rates, intracellular leakage, cell degradation, and poor extraction reproducibility [45–47]. An efficient, robust, and simple yet reproducible method is desirable to reflect the real changes of cellular metabolism [48, 49]. Therefore, optimization of steps involved in the preparation of metabolites is exceptionally significant to ensure that the recovery of metabolites will produce reliable results [50, 51].

Monitoring variability of dataset is essential to ensure that the result is reproducible and reliable. The type of media used to grow the bacteria in the study is crucial in observing variations in the results [52]. Well-controlled processes and conditions of bacteria cultivation can enhance the reproducibility of the results [53]. The use of different culture media, either minimal or rich media such as Mueller-Hinton broth [54], nutrient broth [49, 53, 55], and Luria-Bertani (LB) [56], has been reported in optimizing the bacterial growth conditions for metabolomics studies [52]. Replication of samples, particularly, the biological repeats is important to determine any statistically significant changes of metabolites between the groups [13, 57]. Roberts et al. [21] recommended that the number of replicates can be determined from a power calculation which incorporates baseline biological variability, technical reproducibility, and expected differences. A number of microbial metabolomics studies reported at least three biological replicates in a setup of a sample group [49, 58, 59]. Triplicates or more samples enable the calculation of a relative standard deviation (RSD) value of which less than 20% reflects a well-balanced extraction rate with repeatability in LC-MS analysis [59]. In some metabolomics studies, the reported RSD value can be up to 30% [18, 47, 59, 60]. The output from the analytical process generates large raw datasets which requires a robust, appropriate, and systematic analysis procedure to visualize and identify relevant and meaningful information. The variability of data within and between the sample groups is one of the greatest challenges in analyzing metabolomics datasets [61]. In addition, a quick indicator to test on the reproducibility of the method is by multivariate data analysis including principal component analysis (PCA) and partial least-squares discriminant analysis (PLS-DA) [59].

The choice of analytical platform used for sample analysis depends on the type of metabolites of interest and the sensitivity of the instrument. There is no single analytical instrument that fits all analysis. The most favourable techniques are LC-MS, GC-MS, and NMR, which come with advantages and disadvantages that need to be considered. GC-MS is commonly used in profiling global metabolites with an ability to analyze a large volume of samples and producing high accuracy of metabolites' peak identification [62]. However, an additional step of chemical derivatization in preparing the sample is needed for nonvolatile compounds [62, 63]. In GC-MS, the separation of compounds takes place in a high temperature oven; therefore the compounds need to be thermally stable and volatile [63]. Nevertheless, the additional step of chemical derivatization may cause some loss of metabolites. LC-MS analysis requires no derivatization step and produces high resolution and reproducible results [62]. As for NMR, it is a straight-forward and automated approach with the ability to identify and simultaneously quantify a vast range of organic compounds. However, its poor sensitivity limits its versatility in analyzing large volumes of low-abundance metabolites [62].

## 4. Sample Preparation Methods for Bacterial Metabolites

In metabolomics, one protocol does not fit all. Variations in the physicochemical properties of metabolites (e.g., turnover rates of different metabolite classes) and the biophysical structure of the bacteria themselves (e.g., cell wall and cell membrane permeability) require sample preparation method to be customized and designed accordingly [64]. Optimization of metabolite preparation method would be a prerequisite to study for a particular strain of bacteria or targeted metabolites [56]. An optimized method might not be suitable for a similar bacteria strain implying the different aims and setup of the experiment [65]. Liu et al. [66] highlighted the importance of clear sample preparation method designated for bacterial metabolomics in obtaining high-accuracy and high-reliability data analysis. Most metabolomic studies reported adopting the available or existing procedures conducted for other bacteria or yeast, resulting in inaccurate analysis and conclusion [66]. A number of optimization studies on metabolite sample preparation methods have been reported on various species of bacteria [30, 53, 59]. In addition, several reviews on microbial metabolomics by Mashego et al. [67], van Gulik et al. [68], and Pinu et al. [30] mainly discussed technical aspects, advancements made from the past studies, and future development. Notwithstanding, very little reviews highlighted and discussed the optimized metabolite preparation protocols based on the approaches, either untargeted or targeted.

The diversity of bacterial metabolites and cell wall structure further complicates the sample preparation stage. Due to these factors, different approaches of sample preparation are adopted in maximizing the metabolites obtained. Generally, Gram-positive bacteria require more forces to disrupt their thick cell wall and collecting their intracellular metabolites compared to Gram-negative bacteria [69, 70]. This is due to the presence of rigid and thicker layer of peptidoglycan in the former compared to the latter. The quenching and extraction methods are greatly influenced by the cell wall complexity to ensure that high concentrations of metabolites are collected with minimal metabolite leakages [69, 70]. To date, there have been no specific sample preparation protocols suited for specific types of bacteria. Previous literature which reported on the optimized sample preparation protocols according to the types of bacteria are summarized in Tables 1 and 2.

**Figure 2:**
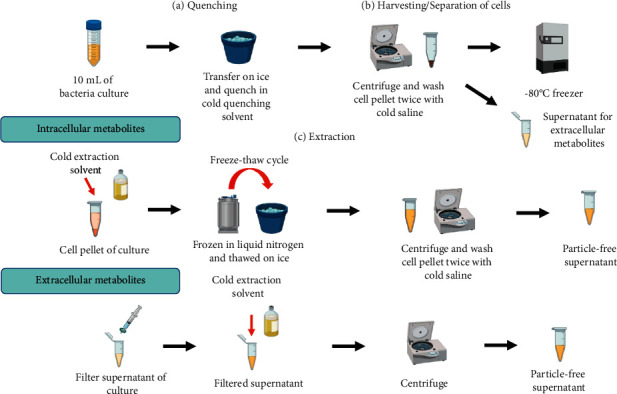
General metabolite sample preparation methods workflow for laboratory scale bacterial metabolomics study. (a) Quenching. (b) Harvesting/separation of cells by centrifugation. (c) Extraction of intra- and extracellular metabolites.

Generally, the process workflow of sample pretreatment method to obtain cellular metabolites includes (1) metabolic arrest by quenching, (2) sample harvesting, a separation of intra- and extracellular metabolites, and (3) metabolite extraction (Figure 2) [35]. Bacteria are grown in an appropriate culture medium to achieve sufficient cell density [52]. Quenching at a specific time and condition is done to stop all the enzymatic and metabolic activities of bacteria cells [55, 66, 71]. Harvesting is to separate the bacterial cells from the culture medium to obtain the intracellular metabolites (from the cell pellet) and extracellular metabolites (from the supernatant) [32]. Finally, extraction is conducted to induce and permeabilize the cells to release metabolites for subsequent analysis.

### 4.1. Sample Harvesting

The steps of harvesting and quenching are often executed concurrently as reported in many literature. Bacterial culture at an optimal density and volume is harvested or collected at a defined time point and condition to ensure that sufficient concentrations of metabolites can be detected [52]. Determination at which time point the sample is harvested is very crucial as cell densities tend to undergo changes between growth phases [52]. In addition, bacteria culture at different point of growth phases produces different types and levels of metabolites, reflecting the different physiological changes within the cell [72, 73]. Between the sample replicates, samples collected shall constitute the same cell density that can be normalized based on the CFU/mL or optical density (e.g., OD600 ∼ 0.5) [52]. Most studies have reported that bacterial samples were commonly harvested in between the early [24, 25, 37, 44] and late exponential growth phases [55].

The harvesting method ideally should be handy and able to effectively separate the cells from the culture media [74]. A study highlighted that the method used on how cells are retrieved accounted for three times higher the total variability compared to the quenching method and extraction solvent selection [59]. Two methods commonly employed for bacterial samples used in a laboratory scale setting either by culture flasks or bioreactors are centrifugation [59, 75] and fast vacuum filtration [52, 75, 76] (Table 3). For centrifugation method, the bacteria culture is subjected to instantaneous quenching and centrifugation together which are often used to analyze all intra- and extracellular metabolites [75]. Centrifugation is more convenient and also helps in reducing variability between sample replicates [52]. Pezzatti et al. [59] reported good repeatability of centrifugation method as indicated by an RSD value of ≤30% with higher abundance of metabolites compared to filtration method. Many studies reported that the duration for centrifugation is between 5 and 8 minutes, which is considered time-consuming and sometimes unsuitable for certain bacteria such as *Staphylococcus aureus* [49, 52, 75]. The longer time taken could induce significant physiological stress to the cells causing metabolite leakage that is likely to compromise the reliability of the analysis [49, 59].

Fast filtration method has been in favour as the process is significantly shorter than the centrifugation method (Table 3) [52, 59, 75, 76]. A membrane filter with an appropriate size is aided by a vacuum system to enhance the speed and separation of bacterial cell from the culture media [77]. The cells deposited on the filter are collected while simultaneously discarding the culture media. The procedure advantageously causes negligible intracellular metabolite leakage as the cells are already being separated from the culture media [44, 49, 56]. Fast filtration immediately after quenching helps to minimize the contact time of the cells with the quenching solvent which prevents massive intracellular leakage [78]. It is important to ensure that the concentration of cells must be at the optimal density to prevent any filter blockage [52]. The removal of adhered cells from the surface of membrane filter paper is also a great challenge. It has been shown that fast filtration produced RSD values of more than 40% portray a deleterious variability in the results [52]. However, filtration method is less suitable for metabolites with high turnover rates such as those in glycolysis and pentose phosphate pathway [77]. An advanced automated filtration system enables a wider range of metabolites to be quantified but requires a specific system development which is a limitation to unskilled researchers [44]. Fast quench is the combination of robust fast filtration system with washing, quenching with solvent, and direct pouring into liquid nitrogen, a less than 30-second process allowing tight preservation of the metabolic state [79].

### 4.2. Quenching

Studies have reported that quenching process can be performed before, during, or after harvesting of sample [36, 49, 58, 80–82]. The step is essential to “quench” or stop or at least slow down the turnover rate and *in vivo* metabolic reactions [30, 67, 83, 84]. Quenching is done by exposing the sample to solvent at either extreme temperatures (cold and hot) [24, 52, 75] or extreme pH conditions (highly acidic or alkaline) [13, 30, 68]. As quenching solvents are likely to affect the membrane structure of the cells, the process must therefore be rapid [67, 68, 85]. The contact time of bacteria cells with quenching solvent should be kept to a minimum as time-dependent leakage may occur [66]. In addition, significant cellular changes may occur due to adherence of bacteria cells to the bottom of a centrifuge tube or surface of filter paper during harvesting or the rapid shift of temperature during quenching [52]. Immediate quenching of bacterial cell for a large sample volume is a great challenge [24]. To date, there has been no exclusive quenching protocol that can stop metabolic activities as some metabolites exhibit a very quick turn over rate.

Cold methanol (−48°C to −20°C) has been commonly used to quench bacterial cells due to its efficiency [30, 58, 71]. de Koning and van Dam were the first to report on the use of 60% cold methanol as the quenching solvent on yeast samples [86] and the method has been widely applied on bacterial samples [59, 71]. Samples of bacterial culture are added directly and rapidly into 60% cold methanol (−48°C) and then centrifuged at low temperature to remove culture broth and quenching solvent [71]. Studies showed that direct quenching into the solvent produced no or little intracellular metabolite leakage [86]. Likewise, studies by Winder et al. [71] and Pezzatti et al. [59] reported good recovery of metabolites from cold methanol quenching. Winder et al. [71] highlighted that pure cold methanol (−48°C) was optimal for *Escherichia coli* as the result produced the highest recovery peak compared to hot ethanol. Quenching with cold methanol: water (8 : 2, −20°C) successfully retrieved abundance of polar metabolites, especially coenzyme A (CoA) and CoA thioester derivatives, citric acid, and some nucleotides in *Caulobacter crescentus* [59]. The use of 60% methanol (−40°C)/0.9% ammonium carbonate (NH_4_HCO_3_) detected about 127 metabolites of *Bacillus licheniformis*, where the salt helped in improving the exudation of metabolites as it reduces the osmotic stress on the cells [87].

Majority of the optimized quenching methods used to process samples for Gram-negative bacteria have adopted the use of cold organic solvents (Table 1). Cold organic quenching solvents can cause up to 60% metabolite leakage as the cold shock and direct contact with the sample could induce permeabilization of cells [80, 88]. Membrane cells are vulnerable to cold methanol, and this has been observed in both untargeted and targeted metabolomics [48, 67, 77, 85, 89, 90]. Wittmann et al. [89] highlighted that although lower methanol concentration was used, the amount of metabolite leakage was about 30%. A quenched *Corynebacterium glutamicum* with buffered methanol (−50°C) demonstrated a significantly lower concentration of free amino acids compared to the unquenched sample [89]. A study by Winder et al. [71] also noted a reduced number of recovery metabolites of E. *coli* such as glutamic acid and putrescine. For global metabolic profiling, the leaked metabolites are mixed with the media components and thus tend to compromise the results [24]. In addition, methanol residual may contaminate the supernatant leading to lyophilization as some leaked metabolites may be lost with volatilized methanol [24]. Cold methanol is also unable to completely halt enzymatic reactions, resulting in high potential of intracellular metabolite changes during quenching which may compromise the overall analysis [30].

Quenching by cold glycerol-saline has been shown to produce better results compared to cold methanol. Villas-Boas and Bruheim [85] reported that cold glycerol-saline (3 : 2) showed much higher intracellular metabolite recovery in *Pseudomonas fluorescens* compared to cold methanol. A similar finding showed that cold glycerol-saline only caused minimal cell damage towards *Lactobacillus paracasei* [84]. Chen et al. [91] demonstrated that the use of methanol/glycerol (−20°C) successfully recovered and identified a high concentration of intracellular metabolites in *Lactobacillus bulgaricus*. In another study, methanol/glycerol (−50°C) was shown to significantly reduce leakage of ATP in *E. coli* compared to methanol/water [92]. Nevertheless, glycerol may also cause lower detection and identification of metabolites as it likely remains and adheres to cells in the supernatant after harvesting [83]. Spura et al. [83] examined the effects of glycerol-saline (−20°C) and 40% ethanol (−20°C) towards *C. glutamicum* and *E. coli* and found that the former took five times longer and the adhered glycerol was difficult to be removed from the pellets. To reduce the glycerol, an additional step of washing with ice-cold 0.9% sodium chloride may be required but it may not be able to remove it effectively [83].

Liquid nitrogen as the quenching agent has been reported in untargeted studies (Table 1) [24, 49, 56]. Notably, Meyer et al. [49, 56] demonstrated that fast filtration prior to liquid nitrogen quenching showed no significant metabolite leakage in *S. aureus* and improved metabolic arrest in *Bacillus subtilis*. Bertini et al. [24] reported that liquid nitrogen quenching of *E. coli* showed less metabolite leakage compared to 60% methanol (−40°C). The results noted that liquid nitrogen has less influence towards cell viability and requires no additional step of lyophilization. Bordag et al. [79] indicated that pouring liquid nitrogen directly onto cells after filtering the washing solution can eliminate the possible time variations. However, it is the least used method due to some drawbacks [24, 49, 56]. The metabolite leakage seems to be unavoidable as liquid nitrogen produced ice crystals which can damage the cell membrane [24].

Quenching ideally should maintain the condition and stability of metabolites [93]. Many studies reported that quenching was performed just after the sample harvesting to minimize metabolite leakage. A few studies indicated that cold methanol quenching in combination with fast filtration produced a reliable metabolite recovery, effective, and highly reproducible results [75, 76, 78]. A study by da Luz et al. [44] reported that an automated fast filtration with on-filter culture with 60% methanol (−45°C) of *E. coli* reduced the total sampling time and metabolite leakage. Aros-Calt et al. [36] also reported the use of filter-based system for simultaneous bacteria isolation and quenching by applying the on-filter system. The filter system containing bacteria was set by having the agar plate loaded with culture medium facing up. The results showed no metabolic disruption of Methicillin-resistant *Staphylococcus aureus* (MRSA) with good reproducibility and consistency [36].

Quenching is crucial to induce metabolic arrest to represent the exact metabolite changes at a particular condition, yet some studies omit the step and this has been observed in both untargeted [25, 53, 72, 94] and targeted studies [38, 95]. Washing the cells upon harvesting and instantaneously immersing into cold extraction solvent are advantageous for high turnover rates of some metabolites to minimize metabolites losses [25, 38, 53, 72]. This is evidence shown in most of the untargeted studies with the highest detection and recovery of high energy metabolites in *E*. *coli* [25, 94]. Sample extraction with no quenching step showed robust and reproducible outcomes with successful detection and identification of metabolic pathways in *S*. *aureus* [72]. Meanwhile, for targeted approach, studies reported that nonquenching sample gave confident detection of metabolites of interest in *E*. *coli* [38] and MRSA [95].

For targeted metabolomics, there was a lack of optimization studies on quenching, particularly with Gram-negative bacteria. Faijes et al. [58] reported that 60% methanol (−40°C) with 0.85% ammonium carbonate only caused less than 10% of metabolite leakage in *L*. *plantarum*. Ammonium carbonate aids by avoiding osmotic shock to the cells and can be removed easily during freeze-drying by evaporation [58]. Lei et al. [82] demonstrated that using a filter-based system with ethanol (−20°C) prior to liquid nitrogen quenching reduced metabolite leakage and produced highest recovery of almost all amino acids in isolating MRSA cell. An optimized quenching for *Streptomyces ZYJ-6* using molar transition energy (ET) showed that isoamylol with base solution of acetone : ethanol (1 : 1) (5 : 1, v/v) at −30°C resulted in the least intracellular metabolite leakage compared to other solvents [66]. The base solution was added to the quenching solution to maintain the membrane's integrity of the cells, yet time-dependent leakage might still occur, highlighting the need to minimize the contact time between the bacterial sample and quenching solution [66].

### 4.3. Extraction

Extraction is the final preparation step to obtain metabolites from the processed sample. The aims of extraction are to deactivate the cellular enzymatic reactions and permeabilize the cells to release metabolites [71]. The method of extraction has a significant impact towards the nature and number of metabolites collected as well as the reproducibility of the study [25]. For untargeted metabolomics, the process should be able to maximize the recovery of a wide range of metabolites of different classes, whereas, in the targeted approach, it is aimed to only extract metabolites of interest [21]. In some cases that require complete metabolome analysis, multiple extraction methods would be necessary to obtain a more comprehensive range of metabolites due to limitations of each method involved [25, 71, 96]. An efficient extraction method generally should be able to (i) disrupt bacterial cell envelop (i.e., cell membrane and cell walls) to release sufficient and desired metabolites, unbiased towards certain physicochemical properties of metabolites, (ii) denature all enzymatic reactions completely, and (iii) prevent any significant chemical conversion and degradation of metabolites [25, 49, 55, 67, 68].

Extraction of metabolites can be performed by chemical, mechanical, or combination of both methods. In this review, a literature summary of the optimized various extraction procedures employed in both untargeted and targeted bacterial metabolomics studies is shown in Table 2. Chemical extraction involves the use of organic solvents: both polar and nonpolar, inorganic nonaqueous, and combinations of both [30]. The choice of extraction solvent depends on a number of factors including the total sample volume, the extraction time, and the coverage of metabolites [96]. The mechanical methods include the use of ultrasonic bath [83], glass bead beating [49, 52, 72, 82], bead mill [53, 87], freeze-thaw [55, 56, 59, 71, 84, 97], and the least favourable, supercritical fluid extraction [98]. Mechanical extraction is commonly applied for animal and plant samples but it is least preferred for microbial intracellular metabolites as the method releases both small and large metabolites [30]. To enhance the efficiency of metabolite extraction, a number of studies have been reported on the combination of chemical and mechanical methods including methanol/ethanol with freeze-thaw cycles [55, 56, 59, 66, 71, 84, 97], methanol/ethanol with bead mill [53], bead milling in liquid nitrogen [87], and glass bead beating [49], as well as sonication with methanol [55] and ethanol [83].

Optimal extraction procedures for both untargeted and targeted bacterial metabolomics mostly reported the use of cold methanol (Table 2) [24, 25, 58, 85, 95, 99]. Cold methanol has been applied for a one-step global metabolite analysis for bacteria samples for quenching and extraction. Methanol can easily evaporate to concentrate the samples without addition of salts, with reproducible results [53]. Cold methanol (−48°C to −40°C) showed the best efficiency of extraction with excellent recovery for most of polar metabolite classes including amino acids, phosphorylated sugars, and nucleotides [25, 59, 71, 85].

Cold methanol has also been used with other solvents such as acidic acetonitrile [94] and chloroform [24, 75] (Table 2). Rabinowitz and Kimball [94] demonstrated that several types of acetonitrile-containing solvent mixtures significantly extracted nucleotide triphosphate with ≥5 times higher yields compared with methanol/water solvent alone. In addition, acidic acetonitrile-methanol desirably minimized the loss of high energy metabolites (e.g., NADPH, CTP, and GTP) and reduced the conversion of the metabolites into low energy derivatives [94]. Moreover, Zhong et al. [38] adopted an optimized methanol: acetonitrile: water with 0.1% formic acid in extracting *E. coli* metabolites which confidently detected 106 isotope-labelled metabolites and quantified 21 isotope-labelled metabolites. The study indicated that a lower freezing point of the solvent is an advantage over acetonitrile: water as it helps in maintaining the cooling effect of quenching cells [38].

Furthermore, the use of chloroform in combination with other solvents has been reported in several studies [24, 37, 75]. Chloroform is used as it can efficiently enhance cell wall disruption and enzyme inactivation [22, 45, 46]. The solvent mixture separates the upper (aqueous) phase and lower (nonaqueous) phase. NMR analysis by Bertini et al. [24] showed that methanol: chloroform extracted majority of the metabolites at a higher concentration including lipids (likely the bacterial membrane lipid and peptidoglycan) compared to cold methanol alone. However, the relatively nonpolar methanol/chloroform mixture was not efficient in extracting for less soluble and highly polar compounds such as sugar phosphates and nucleotides and thus is not suitable for global metabolite profiling [25, 71, 94, 100]. Winder et al. [71] demonstrated that the reduction in the numbers of peaks was probably due to partitioning of the fatty acids and lipids into the chloroform phase compared with the use of methanol alone. Methanol: chloroform: water (3 : 1: 1, v/v) efficiently extracted a wide range of metabolite groups compared to the individual solvents [54]. Moreover, single phase methanol: chloroform: water extraction showed both high recovery and reproducibility for total intracellular lipids in *Pseudomonas aeruginosa* [18].

Ethanol has been used as an extraction solvent in both hot and cold conditions. Boiling ethanol which was used to extract metabolites of *E. coli* helped in stabilizing the metabolites [44, 71]. Besides, boiling ethanol has successfully extracted amino acids and their intermediates. However, the use of hot ethanol is very limited to thermolabile compounds and has low reproducibility [25, 71]. Wordofa et al. [60] adopted boiling ethanol: water (75 : 25, v/v) at 70°C upon fast filtration for targeted analysis of *Pseudomonas taiwanensis VLB120*. The method successfully detected and quantified 107 and 94 metabolites, respectively, including nucleotides, amino acids, central carbon metabolism intermediates, and redox cofactors [60]. In contrast, cold ethanol (−20°C) showed high efficiency and reproducibility, particularly in extracting polar compounds such as phosphorylated sugars [36]. Interestingly, a study by Hiller et al. [101] which reported the use of buffered hot water (95°C) portrayed a good extraction solvent with reliable reproducibility and small detection limits, allowing estimation of true *in vivo* enzyme kinetics of *E*. *coli*.

Strong acids (e.g., perchloric acid and trichloroacetic acid) [58, 67] and strong alkali (e.g., sodium hydroxide and potassium hydroxide) [67] have been shown to cause destruction to metabolites as they could not withstand the acidic and alkali conditions [25]. Maharjan and Ferenci [25] identified lower levels of adenosine and glutathione in both perchloric acid and alkaline extraction methods. In another study, perchloric acid and potassium hydroxide (KOH) extractions yielded low numbers of peaks and very poor reproducibility [71]. Nevertheless, KOH produced unique metabolites in which the majority were short-chain organic acids that were not detected with the methanol method [71]. In acid-base method, an additional neutralization step may be required which is likely to cause a dilution effect and a reduction in metabolite recovery [71]. Otherwise, metabolites may be absorbed into the precipitate and this could affect the metabolite quantification [32].

The combination of chemical and mechanical extraction methods has been commonly adopted in untargeted approach (Table 2). Cold methanol/ethanol or cold methanol: chloroform: water with repeated freeze-thaw cycles has been found to be a favourable method as reported in most literature. Methanol (−48°C) plus freeze-thaw cycles gave the highest peaks of metabolite detection in *E*. *coli* [71] and was favoured for hydrophilic metabolites such as long chain fatty acids in *L*. *paracasei* [84]. The retrieval of important metabolites was the highest (95%) when cold methanol: water with freeze-thaw cycles was applied [59]. A recent study also showed thawing-freezing in cold (−30°C) cryostat and liquid nitrogen in 50% methanol produced a good recovery of most highly abundant intracellular metabolites of *Streptomyces ZYJ-6* [66]. Ethanol alone is not sufficient to break the cell wall of even Gram-positive bacteria which requires an additional step [49]. Ethanol (−20°C) with glass bead has been demonstrated to be more efficient compared to the bead mill, with relatively better metabolite concentrations of *S*. *aureus* [49]. In addition, ethanol (−20°C) with freeze-thaw cycles produced the highest yield of metabolites including organic acids and amino acids, nucleotides, cofactors, and sugar-phosphates in *B*. *subtilis* [56]. Bead milling in cold (−80°C) methanol: water produced higher yield of metabolites with more efficient dispersal of cells compared to ultrasonic bath and was applicable to both Gram-negative and Gram-positive bacteria [53]. Similarly, bead milling in liquid nitrogen showed better identification of *B*. *licheniformis* metabolites than ultrasonic bath as bead milling operated less manually with greater cell disruption rate [87]. Meanwhile, extremely low temperature conditions due to liquid nitrogen maintain the stability of metabolites during the cell disruption process [87]. Bead beating in methanol: chloroform: water has been demonstrated as a fast and reproducible method which allows the comparison of different growth states of *S*. *aureus* [72]. Nevertheless, the rough nature of bead beating could lead to an increase in temperature and cause more degradation of metabolites which would be more suitably used for tissue fractions [59].

## 5. Conclusions

Metabolomics contributes a significant value of data to comprehending a complete biological system of microorganisms through both global metabolic profiling and targeted analysis. A reliable sample pretreatment protocol is essential in metabolomics to understand the fundamental metabolic systems of an organism. The upmost important steps in metabolite preparation are the quenching and extraction, where the methods can significantly impact on selectivity and overall efficiency. However, sample pretreatment method remains a challenge as it highly influences the validity and reliability of a study, which needs further development and improvement. The main challenges in sample preparation for microbial cell cultures are high turnover rates of some metabolites, cell membrane permeability leading to leakage, degradation, poor extraction reproducibility, and cold shock tolerance. The vast variation in bacterial strains and characteristics as well as microbial response towards different solvent types and treatment conditions makes it almost impossible to standardize one method and universal extraction protocol that fits all types of microorganisms.

Quenching the metabolites instantaneously with cold organic solvent by cold shock technique is able to halt majority of metabolite activities and is applicable to a wide range of metabolites. Although metabolite leakage is an ultimate challenge, the condition can be corrected by appropriate analytical techniques and the recovery of metabolites is often sufficient for a reliable analysis. Extracting the metabolites, especially intracellular metabolites, is best performed by the combination of mechanical cell wall disruption and cold solvent extraction. Freeze-thaw cycle in cold solvents such as liquid nitrogen facilitates the breakdown of cell wall peptidoglycan and other biomolecules, enabling maximum release of metabolites. The mixture of polar and nonpolar solvents such as chloroform : methanol : water allows for a wide range of polar and nonpolar metabolite extraction. Nevertheless, exposure to organic solvents and excessive forces might be sensitive for some metabolites.

A careful selection of suitable sample preparation method is crucial, and it is always recommended that optimization of protocols be performed prior to a metabolomics study. The method may not be as specific or perfect, but it would greatly help in excluding contaminations or presence of artefacts that may compromise the results and conclusions. Improvement and optimization are continuously being made by researchers over time to achieve comprehensive extraction protocols that best suit for all types of bacteria. In the future, good and reliable references from optimization of metabolomics studies would be helpful in ruling out the challenges and in further understanding complex and complete systems biology, integrating all the high-throughput data.

## Figures and Tables

**Table 1:** Summary of optimized quenching methods in bacterial metabolomics studies.

Bacteria	Approach	Optimal quenching method	Findings	Ref.
Gram-negative				
*Saccharophagus degradans*	Untargeted	(−70°C, 70%) methanol	(i) Severe cell leakage induced significant loss of intracellular metabolites.	[90]
*E*. *coli*	Untargeted	(−48°C, 60%) methanol	(i) Produce the highest recovery of intracellular metabolites with highest peak of metabolites detected.	[71]
*E*. *coli*	Untargeted	Automated fast filtration with on-filter (−45°C, 60%) methanol	(i) Significantly higher intracellular concentrations of amino acids were obtained.	[44]
			(ii) Minimize total sampling time and metabolite leakage.	
*E*. *coli*	Untargeted	(−50°C) 60% methanol/40% glycerol	(i) Methanol/glycerol significantly reduced leakage of ATP (15–16%) compared to 60% methanol.	[92]
*E*. *coli*	Untargeted	Liquid nitrogen	(i) Enhanced metabolites recovery compared to 60% methanol (−40°C) that caused more metabolites leakage.	[24]
Gram-positive				
*B*. *licheniformis*	Untargeted	(−40°C) 60% methanol/0.9% NH_4_HCO_3_	(i) Detection of 127 metabolites with vast amount of amino acids, organic acids, and carbohydrates.	[87]
			(ii) Improved protein exudation and reduced metabolites leakage.	
			(iii) NH_4_HCO_3_ is suitable for LCMS requirements of metabolomics analysis.	
*C*. *crescentus*	Untargeted	(−20°C, 80%) methanol	(i) Higher recovery of polar compound including CoA and CoA thioester derivatives, citric acid, and some nucleotides.	[59]
*P*. *fluorescens*	Untargeted	Cold glycerol-saline (3 : 2), glycerol-water (3 : 2), glycerol-mannitol (3 : 2)	(i) Glycerol-saline (−23°C) produced higher detection and less metabolite leakage compared to cold methanol.	[85]
*L*. *bulgaricus*	Untargeted	(−20°C, 80%) methanol: glycerol	(i) The solvent applicable to other Gram-positive bacteria *(S. coelicolor)* and yeast *(S. cerevisiae)*.	[91]
MRSA	Untargeted	On-filter culture (20°C, 60%) ethanol	(i) No significant metabolic disruption.	[36]
			(ii) Good reproducibility and consistency.	
*S*. *aureus*	Untargeted	Fast filtration followed by (−20°C, 0%) ethanol and liquid nitrogen	(i) Separation of cells prior to quenching caused no significant metabolites leakage with better energy charge.	[49]
			(ii) Effective quenching is achieved by (−20°C, 60%) ethanol.	
*B*. *subtilis*	Untargeted	Liquid nitrogen with vacuum filtration	(i) Improved metabolic arrest during filtration.	[56]
*C*. *glutamicum*, *E*. *coli*	Untargeted	(−20°C, 40%) ethanol and 0.8% (w/v) sodium chloride	(i) Highest detection and identification of metabolites with ethanol quenching (118 metabolites) compared to 60% methanol (−50°C) and glycerol-saline (−20°C).	[83]
*Lactobacillus plantarum*	Targeted	(−40°C, 60%) methanol with 0.85% ammonium carbonate	(i) 60% methanol (−40°C), 60% methanol (−40°C)/ 0.85% NaCl/HEPES (70 mM) showed more than 10% cell leakage.	[58]
MRSA	Targeted	Filter-based system with (−20°C) ethanol plus liquid nitrogen	(i) Highest recovery of almost all amino acids.	[82]
			(ii) Reduced metabolites leakage.	
*Streptomyces ZYJ-6*	Targeted	Isoamylol: (acetone: ethanol, 1 : 1) (5 : 1, v/v)	(i) 60% methanol produced the largest metabolite leakage, followed by acetone: base, methanol: base, and propanol: base.	[66]

**Table 2:** Summary of optimized extraction methods for bacterial metabolomics studies.

Bacteria	Approach	Optimal extraction method	Findings	Ref.
Gram-negative				
*E*. *coli*	Untargeted	(−40^o^C) methanol	(i) Highest detection of metabolites spots (80–99) compared to perchloric acid, alkaline, hot ethanol, methanol/chloroform, and hot methanol.	[25]
*E*. *coli*	Untargeted	(−48°C) methanol plus freeze-thaw	(i) Highest recovery of peaks from methanol extraction method compared to other methods.	[71]
*E*. *coli*	Untargeted	Acidic acetonitrile-methanol	(i) Extraction minimizes the loss of high-energy metabolites and their conversion into low-energy derivatives.	[94]
*E*. *coli*	Untargeted	(−40°C) methanol: chloroform (1 : 1)	(i) (−40°C) methanol: chloroform (1 : 1) extracts higher concentration of metabolites compared to (−40°C) methanol.	[24]
*E*. *coli*	Untargeted	Buffered hot water (95°C)	(i) Buffered hot water showed the best reproducibility with smallest detection limits that enable estimation of true *in vivo* enzymes as exemplified for fructose 1,6-biphosphate, and citrate synthase.	[101]
*C*. *crescentus*	Untargeted	(−20°C, 80%) methanol: water (8 : 2) with freeze-thaw cycles	(i) High recovery of polar metabolites, CoA and CoA thioester derivatives, citric acid, and some nucleotides.	[59]
*E*. *coli, P*. *aeruginosa,* S. *typhimurium*, and *MSSA*	Untargeted	Bead milling in (−80°C) methanol: water (9 : 1)	(i) Higher yield of metabolites with more efficient dispersal of cell pellet.	[53]
*P*. *taiwanensis VLB120*	Targeted	Pressure driven fast filtration approach followed by boiling ethanol: water (75 : 25, v/v) at 70°C	(i) Detection of 107 metabolites and quantification of 94 metabolites including nucleotides, amino acids, central carbon metabolism intermediates, and redox cofactors.	[60]
*E*. *coli*	Targeted	40 : 40 : 20 methanol: acetonitrile: H_2_O with 0.1% formic acid	(i) 106°C metabolites were confidently detected and 21 isotope-labelled metabolites were quantified.	[38]

Gram-positive				
MRSA	Untargeted	(−20°C, 60%) ethanol	(i) High efficiency and reproducibility in extracting some polar compounds such as nucleotides and phosphorylated sugar.	[36]
			(ii) Successfully characterized 210 of well-defined compounds.	
*S*. *aureus*	Untargeted	(−20°C, 60%) ethanol plus glass bead with two cycles in homogenizer	(i) Produce the most useful outcome for a global metabolomics analysis with detection of higher concentration and highest number of metabolites.	[49]
*B*. *subtilis*	Untargeted	Two-step extraction method, first with 60% cold ethanol and second with cold water with freeze-thaw	(i) Detection of highest metabolite amounts with a good EC-value.	[56]
*S*. *aureus*	Untargeted	Bead beating in a cold (−20°C) methanol: chloroform: water (3 : 1: 1)	(i) Fast and reproducible, allows direct comparison between different bacterial growth states.	[72]
*B*. *licheniformis*	Untargeted	Bead milling in liquid nitrogen	(i) Identification of 116 metabolites.	[87]
			(ii) More types of amino acids with high concentrations were identified compared to liquid nitrogen grinding.	
*Streptomyces ZYJ-6*	Targeted	Suspension in 50% (v/v) methanol and three cycles of freeze-thaw	(i) 44 of most highly abundant intracellular metabolites were found and quantified.	[66]

**Table 3:** Summary on comparison between centrifugation and fast filtration method for cell harvesting.

	Centrifugation	Fast filtration
Feasibility	(i) More convenient.	(i) Fast processing rate.
	(ii) Reduce variability between sample.	(ii) No or very minimal metabolite leakage.
	(iii) Can be at high cell concentration.	(iii) Instantaneous removal of culture media.
	(iv) Can be subjected to instantaneous quenching	

Disadvantages	(i) Longer processing time.	(i) Only at low cell concentration to avoid filter blockage.
	(ii) May induce physiological stress and metabolites leakage.	(ii) Unsuitable for high turnover rate metabolites.
	(iii) Unsuitable for certain bacteria, e.g., S. *aureus.*	(iii) Requires optimization step.
		(iv) High variability of extraction variation.

## Data Availability

The data supporting this article review are from previously reported studies and datasets, which have been cited.
